# Artificial Intelligence‐Driven Soft Bioelectronics for Self‐Powered Respiration Monitoring

**DOI:** 10.1002/advs.202519271

**Published:** 2026-01-04

**Authors:** Xinkai Xu, Xiao Xiao, Rui Guo, Jun Chen

**Affiliations:** ^1^ Department of Bioengineering University of California, Los Angeles Los Angeles California USA

**Keywords:** artificial intelligence, magnetoelasticity, piezoelectricity, respiration monitoring, self‐powered, triboelectricity

## Abstract

Respiration is a critical physiological process that reflects the health status of the human body. Self‐powered bioelectronic devices for respiration monitoring have shown great promise, driven by their advantages in miniaturization, cost‐effectiveness, high sensitivity, and excellent reliability. This work examines the recent advances in artificial intelligence‐driven, self‐powered respiration monitoring sensors based on triboelectricity, piezoelectricity, and magnetoelasticity, with a focus on their sensing performance and signal transduction mechanisms. A comparative analysis of their performance characteristics and applicable scenarios is presented, together with a discussion of practical considerations including breathability, wearing comfort, and waterproof performance, and an overview of the relative performance and application suitability of the three technologies. Furthermore, this report envisions future directions including long‐term multi‐scenario data collection and big data‐driven respiratory diseases diagnostics. We believe that the widespread implementation of artificial intelligence‐driven, self‐powered respiration monitoring sensors will play a pivotal role in reshaping healthcare and advancing intelligent interventions for respiratory diseases, ultimately promoting global health and well‐being.

## Graphical abstract

Artificial intelligence‐driven soft bioelectronics for self powered respiration monitoring based on triboelectric nanogenerators (TENGs), piezoelectric nanogenerators (PENGs), and magnetoelastic generators (MEGs) enable continuous and multi‐scenario respiratory biomechanical data collection. Coupled with machine learning and big data driven diagnostics, these systems support disease identification, personalized therapy, and responsive intervention.

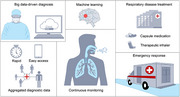

## 1 Introduction

**FIGURE 1:**
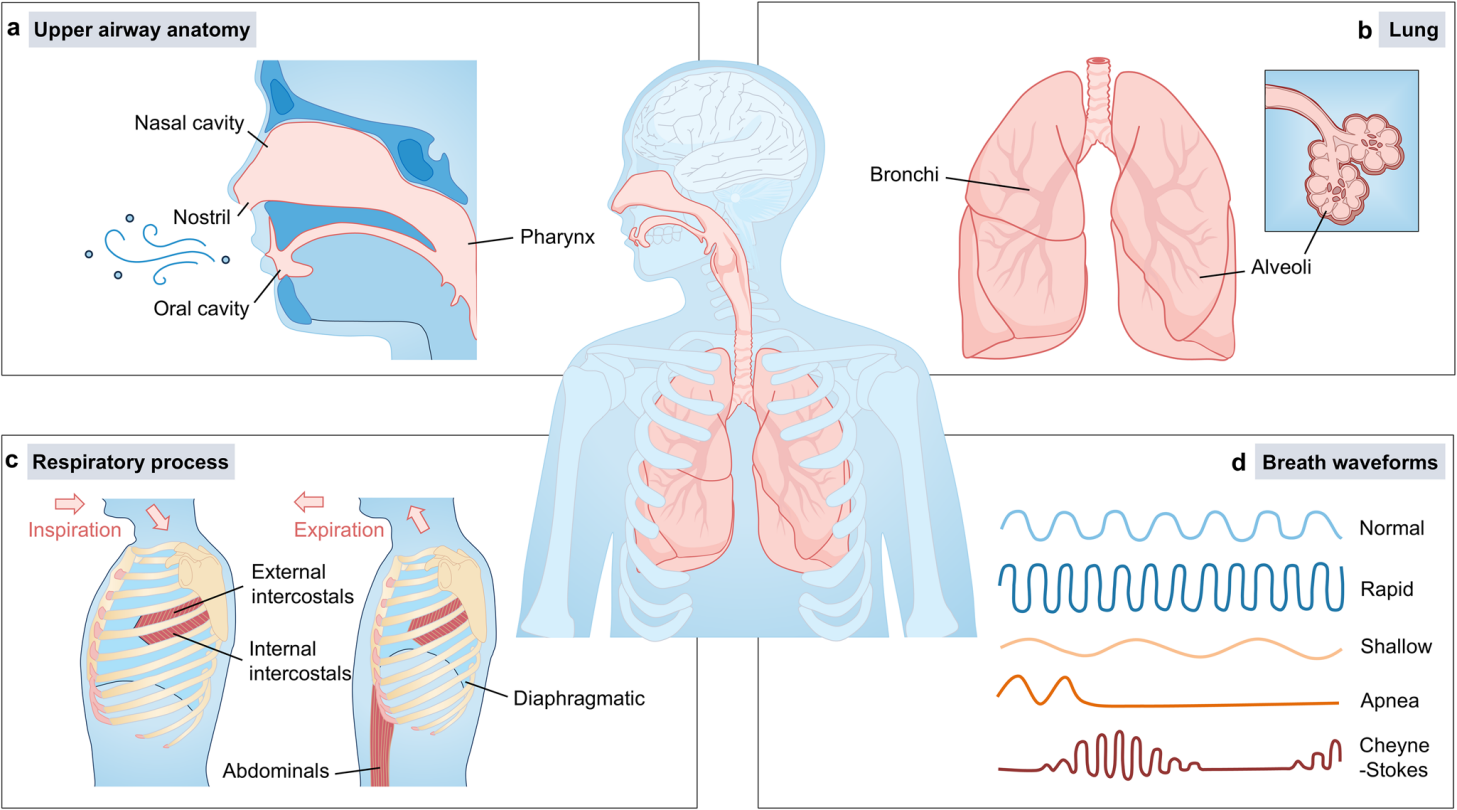
Biological basis of respiration and breath waveforms. (a) Schematic diagram of the upper respiratory tract, including the nostrils, nasal cavity, and pharynx. Air can enter the nasal cavity through the nostrils or pass through the oral cavity into the oropharynx, then travel down the trachea into the lungs. (b) Lung and alveoli. In the lungs, air passes through the bronchi into the alveoli, which are the key sites for gas exchange, delivering oxygen to the bloodstream and removing carbon dioxide. (c) Respiratory process illustration. Respiration consists of two phases: inspiration and expiration. During inspiration, the diaphragm and external intercostals contract to expand the thoracic cavity, allowing air to enter the lungs. During expiration, the internal intercostals and abdominals contract to reduce thoracic volume, expelling air from the lungs. (d) Breath waveforms at different rates and patterns, including normal breathing, rapid breathing, shallow breathing, apnea, and Cheyne–Stokes respiration [12]. Adapted with permission from ref. [12]. Copyright 2023 ELSEVIER.

Catalyzed by the COVID‐19 pandemic and the artificial intelligence (AI), wearable respiration monitoring has attracted significant research attention, highlighting its clinical and societal relevance, with innovative monitoring technologies continually emerging. Respiration is a vital physiological process essential for sustaining human life, primarily responsible for facilitating gas exchange. Inhaled oxygen is absorbed by red blood cells and transported through the circulatory system to tissues throughout the body, where it supports aerobic respiration and cellular energy production [1, 2, 3]. During this process, multiple organs including the upper respiratory tract, trachea, and lungs coordinate to ensure efficient gas exchange [4, 5, 6], as illustrated in Figure 1. Specifically, air enters the body either through the nostrils into the nasal cavity or through the mouth into the oropharynx (Figure 1a), and subsequently travels down the trachea into the lungs [7]. Once in the lungs, air first reaches the left and right primary bronchi, proceeds into progressively smaller bronchioles, and finally arrives at the alveoli (Figure 1b), where oxygen diffuses into the bloodstream to complete the transport process [8]. This respiratory process relies not only on the proper function of associated organs but also on the involvement of muscles and the nervous system. Under the control of the central nervous system, the diaphragm and external intercostal muscles contract to expand the thoracic cavity and draw air into the lungs. During exhalation, the internal intercostal muscles and abdominal muscles contract to reduce thoracic volume and expel air from the lungs, as shown in Figure 1c. The rhythmic contraction of respiratory muscles causes periodic changes in thoracic volume, which in turn drives the rhythmic expansion and contraction of the lungs [9, 10].

Respiration is influenced by all neural, organ‐based, and muscular components involved in the breathing process, making it a key physiological parameter for early detection and continuous monitoring of respiratory diseases [11]. The monitored respiratory patterns can be categorized into various types, including normal, rapid, shallow, apnea, and Cheyne–Stokes respiration (Figure 1d) [12]. These patterns play significant roles in different pathological conditions. For example, in restrictive lung diseases, respiration monitoring helps assess the degree of airway obstruction and overall deterioration of respiratory function in patients with pulmonary fibrosis, thereby assisting physicians in adjusting treatment strategies and identifying severe complications such as acute respiratory failure in a timely manner [13]. In the context of sleep‐related breathing disorders, particularly obstructive sleep apnea, patients may suffer from significant upper airway obstruction that requires invasive interventions such as maxillomandibular advancement surgery to expand the skeletal framework of the upper airway [14]. Continuous respiration monitoring can detect episodes of apnea or decreased respiratory frequency, enabling timely interventions and treatments for patients [15]. In infectious respiratory diseases, respiration monitoring is equally important for the diagnosis and management of conditions such as pneumonia and tuberculosis. For instance, by evaluating respiratory rate alongside other vital signs such as blood pressure, clinicians can assess pneumonia severity and triage patients accordingly to deliver optimal care [16]. Notably, during recent outbreaks of COVID‐19, routine respiration monitoringhas proven beneficial for evaluating disease progression and enabling early intervention, thereby reducing the risk of complications [17].

To ensure accurate diagnosis and timely treatment of respiratory diseases, biosensors for respiration monitoring must provide continuous, long‐duration operation to deliver accurate and uninterrupted physiological data [18, 19, 20]. However, to maintain portability and user convenience, these sensors are often miniaturized, which inherently conflicts with the demand for long‐term operation due to limited energy supply. To overcome this challenge and meet the growing need for efficient and user‐friendly long‐term monitoring, self‐powered respiratory biosensors offer a highly promising solution. By leveraging physical transduction mechanisms such as triboelectricity [21, 22, 23], piezoelectricity [24, 25, 26], and magnetoelasticity [27, 28, 29, 30], in conjunction with pressure sensors including resistive [31, 32, 33] and capacitive types [34, 35, 36, 37], these self‐powered sensors can be attached or worn on the mouth, chest, or abdomen to continuously monitor respiratory activity with high precision and sensitivity [38, 39, 40]. These systems not only offer low cost and compact size, but also provide reliable respiratory data in real time, supporting clinical diagnosis and treatment for both patients and the general population. Moreover, with the increasing integration of highly economically available materials such as textiles and papers into respiration monitoring sensors [41], as well as advancements in newly developed intrinsically waterproof magnetoelastic generator technologies [29], significant progress has been made in improving user comfort and expanding the applicability of these sensors across diverse real‐world environments.

In this Review, we present a comprehensive analysis of AI‐powered, self‐sustained bioelectronics for respiration monitoring. These devices combine continuous, real‐time respiratory sensing with advanced machine learning algorithms, enabling automated detection, classification, and prediction of respiratory abnormalities. By integrating predictive analytics, these systems can anticipate disease progression and support timely, personalized interventions, thereby reducing both incidence and risk of deterioration. We also examine recent advances in AI‐enhanced functional materials, energy‐efficient designs, and intelligent deployment scenarios. Finally, we highlight the convergence of self‐powered respiratory sensors with the Internet of Things (IoT), focusing on AI‐driven big data analytics, historical health data integration, and decision‐support frameworks that facilitate precise, proactive management of respiratory health.

## 2 Platform Technologies

**FIGURE 2:**
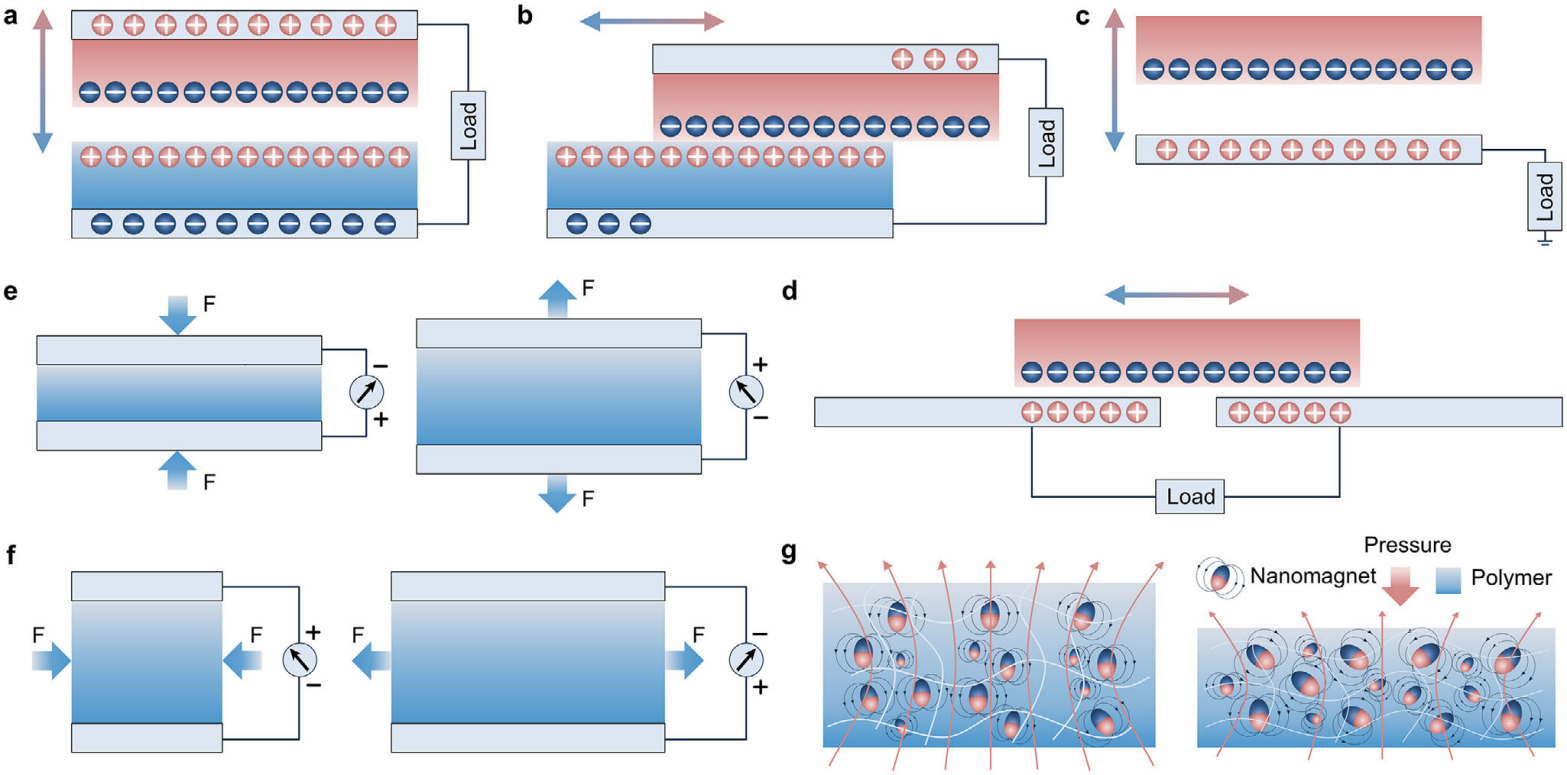
Working principles of the generators for self‐powered respiratory rate monitoring. (a) Triboelectric nanogenerators (TENGs) vertical contact‐separation working mode. Two dielectric films with different triboelectric properties contact face‐to‐face, generating charges. Separation produces a potential difference that drives current. (b) TENGs lateral‐sliding working mode. Two dielectric films slide parallel to the contact interface, generating charges and potential that drive current. (c) TENGs single electrode working mode. The bottom surface acts as a reference electrode to harvest energy from freely moving objects. (d) TENGs freestanding triboelectric layer working mode. Symmetric electrodes sense the asymmetric charge distribution of a freely moving object, inducing current between electrodes [43]. Adapted with permission from ref. [43]. Copyright 2014 RSC Publishing. (e) Piezoelectric nanogenerators (PENGs) d_33_ working mode. Using piezoelectric materials as electrodes, the polarization direction is parallel to the applied stress. Pressure applied along the polarization direction generates potential. (f) PENGs d_31_ working mode. The polarization direction is perpendicular to the stress direction. Vertical strain induced by external force generates potential along the polarization direction [48]. Adapted with permission from ref. [48]. Copyright 2022 IOP Publishing. (g) Magnetoelastic generators (MEGs) working mode. Nanomagnets form a wavy chain structure in a porous matrix. When the elastic matrix is compressed, changes in Nanomagnet spacing and dipole alignment alter the magnetic field [27]. Adapted with permission from ref. [27]. Copyright 2021 Springer Nature.

Currently, self‐powered bioelectronics for respiration monitoring primarily rely on triboelectric nanogenerators (TENGs), piezoelectric nanogenerators (PENGs), and magnetoelastic generators (MEGs). TENGs have attracted considerable attention due to their rapid response time and high sensitivity. Since the first report in 2012, the TENG research field has undergone rapid expansion and has since emerged as an independent research direction [42]. Over time, four operation modes of TENGs have been proposed, including the vertical contact‐separation mode, lateral‐sliding mode, single‐electrode mode, and freestanding triboelectric‐layer mode [43], as illustrated in Figures 2a–d. In the vertical contact‐separation mode, two dielectric films with different triboelectric properties are placed face to face, generating opposite surface charges upon physical contact. When the external force separates the films, the resulting change in gap distance induces a potential difference that drives the current between electrodes (Figure 2a) [44]. The lateral‐sliding mode also utilizes two dielectric films but involves relative motion parallel to the contact interface, generating frictional charges and a lateral potential gradient that facilitates current flow (Figure 2b) [45]. This configuration supports compact encapsulation. In the single‐electrode mode, the bottom surface serves as a reference electrode, allowing energy harvesting from free‐moving objects, such as a human foot interacting with the ground (Figure 2c) [43]. The freestanding triboelectric‐layer mode operates under a similar principle but replaces the grounded electrode with a pair of symmetric electrodes. Asymmetric charge distribution during the motion of a freestanding object induces a current between electrodes (Figure 2d) [46]. These four operation modes are often hybridized in practical applications to achieve enhanced energy‐harvesting performance tailored to specific scenarios.

In contrast to TENGs, which rely on triboelectric effects and electrostatic induction to generate charges, PENGs are devices that utilize piezoelectric materials as electrodes. They generate a potential difference and release charges by breaking the symmetry of the crystal structure under external force [47]. Piezoelectric materials have two main working modes: the d_33_ mode (longitudinal mode) and the d_31_ mode (transverse mode). In the d_33_ mode, the polarization direction is parallel to the applied stress direction. When pressure is applied along the polarization direction, the material generates a potential in the same direction, achieving a higher voltage output (Figure 2e) [48]. In the d_31_ mode, the polarization direction is perpendicular to the direction of stress (Figure 2f). The vertical strain induced by the external force induces a potential in the polarization direction. Although the voltage output is relatively low, this mode is suitable for more flexible device structure designs [49]. Generally, the d_33_ value is greater than the d_31_ value, and in this mode, a larger voltage output can be generated [50]. Therefore, the d_33_ mode is more commonly used in applications that involve piezoelectric energy harvesting.

Although TENGs and PENGs offer advantages such as high sensitivity, adequate breathability, and conformability, the sweat on the skin surface and water contained in the breath can largely affect their output performance and significantly impact their stability. This phenomenon arises from the corrosion of the TENGs friction layer caused by increased humidity, which accelerates charge dissipation, and the reduction of the piezoelectric coefficient in PENGs due to water molecules infiltrating the piezoelectric material [51, 52]. The MEG was first reported by Jun Chen Group in 2021, elucidating a giant magnetoelastic effect in soft magnetoelastic composites and initiating the development of this new class of self‐powered devices [27]. This pioneering work positioned MEGs as a representative and rapidly expanding branch in self‐powered bioelectronics, with subsequent research establishing their strong waterproof properties owing to the magnetoelastic mechanism [30]. MEG is composed of nanomagnets dispersed in a porous silicone rubber matrix. After impulsed magnetization, the nanomagnets form a wavy chain‐like structure, as shown in Figure 2g [27]. When the elastic matrix is subjected to compression, the spacing between the nanomagnets and their dipole alignment change, thereby altering the magnetic field generated by the composite. MEG‐based bioelectronics are flexible, stretchable, and capable of wireless, self‐powered operation. Their functionality in wet environments is enabled by the ability of magnetic fields to penetrate water with negligible intensity loss, ensuring reliable performance and complete waterproofing [28]. The MEGs significantly fill the gap in the field of waterproof bioelectronics, expanding its range of applications.

## 3 Respiration Monitoring via Triboelectricity

By leveraging the surface‐charging effect between dissimilar materials, the soft triboelectric bioelectronics combine softness, lightweight design, biocompatibility, and wearability—even supporting implantable applications—paving the way for transformative advances in soft bioelectronics for personalized healthcare [53, 54, 55, 56]. In the context of respiratory rate monitoring, TENG systems are generally classified into film and fiber configurations. Film‐based TENGs are typically mounted on the chest or integrated into face masks, where they generate electrical signals through contact–separation motions induced by thoracic movements or exhaled airflow [57, 58]. In contrast, fiber‐shaped TENGs are constructed from textile or fiber materials and integrated into wearable garments via weaving or stitching techniques, offering superior flexibility and ease of integration for long‐term, real‐time respiratory monitoring applications [38, 59].

### 3.1 Film‐Based Triboelectric Nanogenerators

**FIGURE 3:**
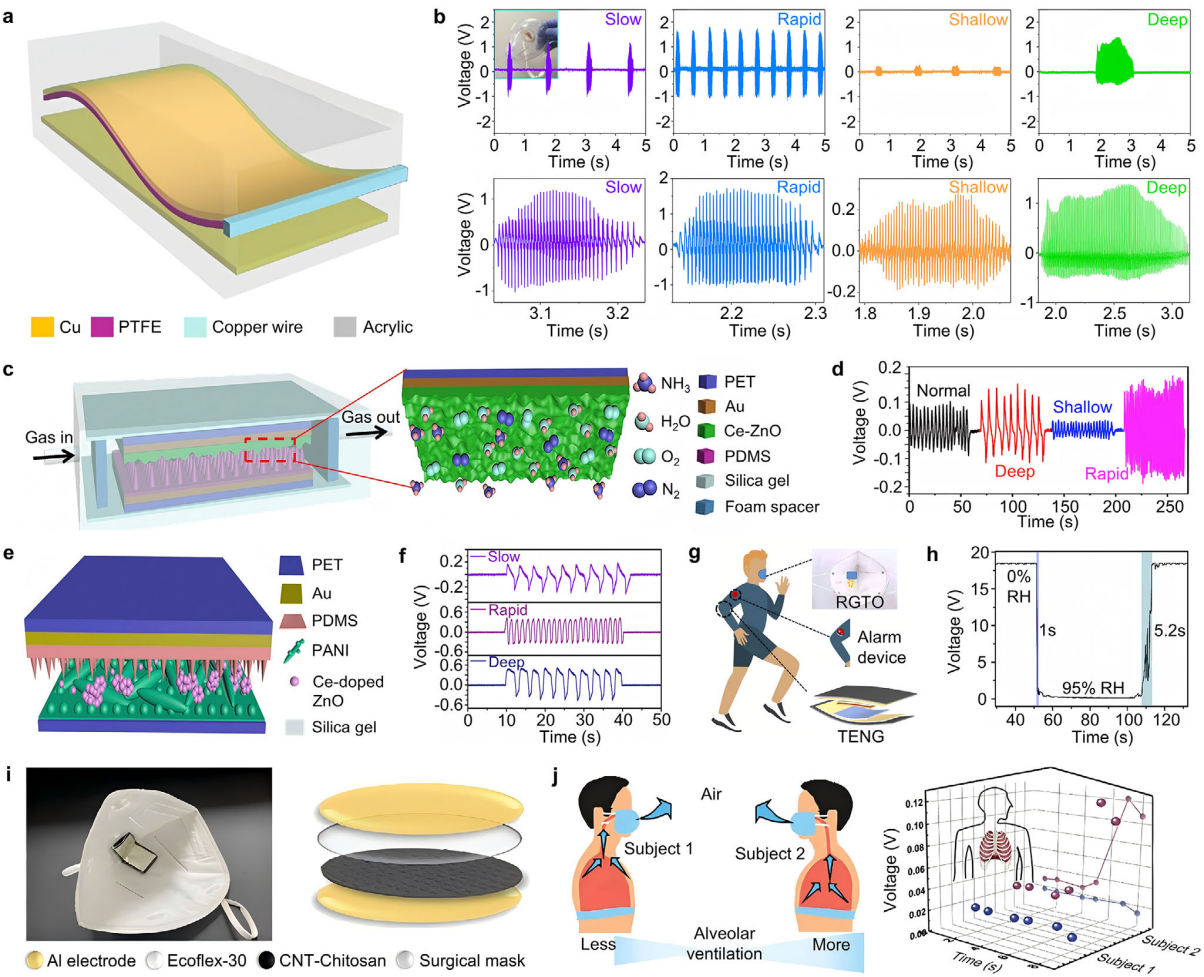
Applications of film‐based triboelectric nanogenerators in respiratory rate monitoring. (a) Schematic configuration of the air‐flow‐driven TENG. (b) Real‐time respiratory signals of the TENG, which were recorded from four different human breathing behaviors including slow, rapid, shallow, and deep breathing [64]. Adapted with permission from ref. [64]. Copyright 2018 ACS. (c) Diagram of the as‐developed triboelectric self‐powered respiration sensor (TSRS) and enlarged view of the Ce‐doped ZnO sensitive layer. (d) Output voltage of the TSRS under four different breathing patterns [66]. Adapted with permission from ref. [66]. Copyright 2019 ELSEVIER. (e) Respiration‐driven self‐powered sensing system. (f) Real‐time respiratory signals of the TENG under three different human breathing behaviors without restricting the respiratory flow [67]. Adapted with permission from ref. [67]. Copyright 2019 ELSEVIER. (g) Respiration monitoring alarm device based on TENG‐driven self‐powered RGO‐TiO_2_ (TENG‐RGTO) sensor. (h) Response and recovery time of TENG‐RGTO sensor [57]. Adapted with permission from ref. [57]. Copyright 2022 ELSEVIER. (i) Ecoflex‐graphene TENG (RC‐TENG) mounted on the inside of the mouthpiece. (j) Sports respiratory state analysis of two subjects [58]. Adapted with permission from ref. [58]. Copyright 2024 Wiley‐VCH.

While conventional real‐time respiratory monitoring devices are often hindered by complex structures, large size, and high cost [60, 61], TENGs, as an emerging technology for energy harvesting and signal transduction, overcome these limitations and have attracted considerable attention [23, 62, 63]. As shown in Figure 3a, a flexible TENG driven by exhaled airflow is capable of generating electrical signals corresponding to various respiratory states, enabling self‐powered respiration monitoring without the need for an external power supply [64]. This flexible TENG is fabricated using a nanostructured polytetrafluoroethylene (n‐PTFE) film coated with an ultra‐thin copper layer for charge collection. One end of the film is fixed at the midpoint of an acrylic tube, with a copper electrode at the base. During operation, the incoming airflow drives the PTFE film into oscillation, resulting in periodic contact and separation with the copper electrode. The n‐PTFE film consists of high‐porosity nanofibers with a large surface area, which facilitates charge transfer and airflow‐driven motion, thereby enhancing the triboelectric output performance [65]. The TENG based on the n‐PTFE film is highly sensitive to respiratory airflow, converting exhalation into electrical signals for real‐time respiration pattern monitoring. When integrated into a medical mask, it passively detects exhaled airflow and generates electrical signals accordingly. Distinct voltage envelopes are produced for slow, fast, shallow, and deep breathing (Figure 3b), allowing accurate differentiation and recognition of respiratory states [64]. Beyond respiration rate monitoring, a triboelectric self‐powered respiration sensor (TSRS) has been developed to simultaneously monitor breathing patterns and ammonia (NH_3_) concentration in exhaled air [66]. As shown in Figure 3c, the TSRS comprises Ce‐doped ZnO, polydimethylsiloxane (PDMS), gold foil, and a PET substrate. The Ce‐doped ZnO serves dual roles as both a gas‐sensing and triboelectric material, while the PDMS functions as the triboelectric layer, backed by a gold electrode. The entire device is encapsulated within a flexible silicone membrane to enhance flexibility and airtightness. The TSRS captures respiratory behavior through the expansion and contraction of the chest. Figure 3d presents real‐time voltage outputs for various breathing patterns including normal, deep, shallow, and rapid breathing. Both frequency and amplitude of the signal allow clear differentiation of respiratory rate and depth, with deep breathing showing higher amplitude and longer intervals than normal breathing [66]. Another respiration‐driven TENG based on a Ce‐doped ZnO‐PANI composite film is also capable of converting respiratory airflow into electrical signals for simultaneous monitoring of NH_3_ concentration, flow rate, and respiratory frequency (Figure 3e) [67]. The PET substrate, electrodes, triboelectric layer, and NH_3_‐sensitive layer are integrated into a single device, in which periodic pressure generated by an elastic balloon drives the contact‐separation motion of the triboelectric layer, enabling self‐powered signal output and gas sensing. As shown in Figure 3f, the device clearly distinguishes between slow breathing (0.3 Hz, 0.46 V), rapid breathing (0.77 Hz, 0.71 V), and deep breathing (0.94 V) [67]. The results align well with actual respiratory behavior [64].

TENGs can also be integrated with other sensors to enable multifunctional applications. For example, a self‐powered humidity sensor composed of reduced graphene oxide (RGO) and TiO_2_ was developed by coupling TENG with humidity sensing elements [57]. This sensor not only measures ambient humidity but also distinguishes respiratory patterns of varying frequency and intensity, enabling a human‐driven respiratory alarm function, as illustrated in Figure 3g. It exhibits a high response of 359.2, fast response/recovery times of 1/5.2 s, and low hysteresis (0.54%) across a wide humidity range of 0%–95% relative humidity (RH) (Figure 3h) [57]. The hydroxyl groups and oxygen vacancies on the surface of TiO_2_ facilitate water molecule adsorption and dissociation, thereby enhancing response speed [68, 69]. These features offer new strategies for designing multifunctional, self‐powered respiratory monitoring sensors. On the other hand, TENGs can also be combined with biodegradable technologies. A wireless intelligent respiratory monitoring system (WIRMS) based on a degradable chitosan‐CNT film and a raised shape Ecoflex‐graphene TENG (RC‐TENG) has been developed, as shown in Figure 3i [58]. The RC‐TENG is mounted using a simple patch strategy, ensuring good wearability, and is capable of real‐time monitoring and diagnosing respiratory disorders during exercise. By analyzing changes in respiratory rate and intensity, the system can evaluate respiratory muscle fatigue (Figure 3j) [58], allowing timely intervention to prevent deterioration when respiratory intensity drops too low [70]. This highlights the promising potential of WIRMS for wearable applications in sports and medical monitoring.

### 3.2 Fiber‐Shaped Triboelectric Nanogenerators

**FIGURE 4:**
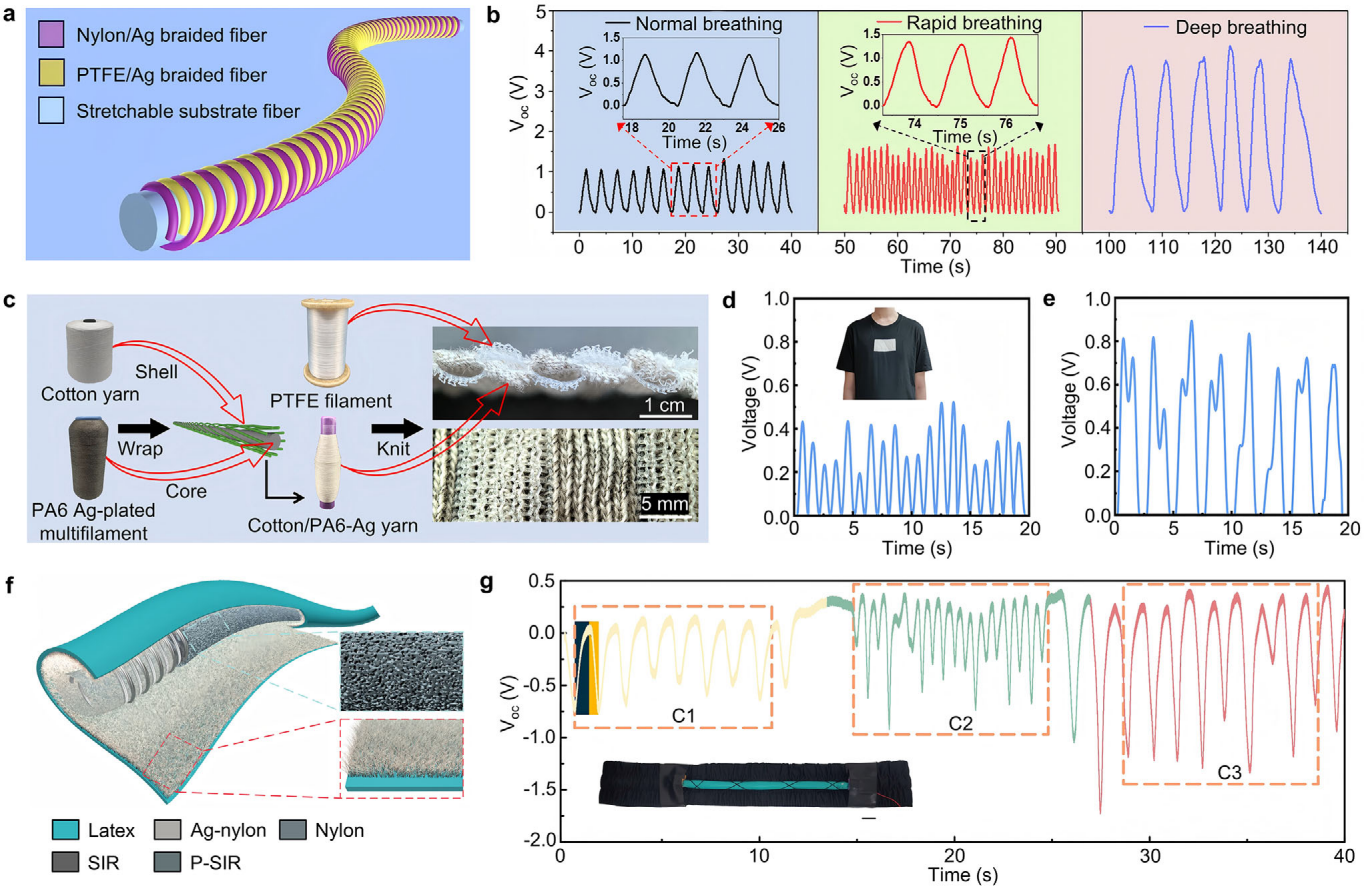
Applications of fiber‐shaped triboelectric nanogenerators in respiratory rate monitoring. (a) Schematic structure diagram of the helical fiber strain sensor (HFSS) with helical structure. (b) Voltage signals recorded normal, rapid, and deep breathing states, and the insets show the enlarged curves [59]. Adapted with permission from ref. [59]. Copyright 2022 ACS. (c) Yarn preparation and cross‐sectional and top‐down views of the interlock stitch knitted TENG (ISK‐TENG) structure. (d) Rapid breathing monitored by ISK‐TENG. (e) Slow breathing monitored by ISK‐TENG [40]. Adapted with permission from ref. [40]. Copyright 2025 ACS. (f) The schematic of the structure of the fish lateral line system fiber‐based TENG (FLLF‐TENG). (g) Digital photograph of the FLLF‐TENG integrated with an elastic band and the voltage output characteristics of the FLLF‐TENG under different breathing modes [38]. Adapted with permission from ref. [38]. Copyright 2024 ELSEVIER.

As another type of TENG with the same principle but a different structure, the fiber‐shaped triboelectric generator (FS‐TENG) has gained widespread attention for its high flexibility and comfortable wearability. Due to the properties of fibers, FS‐TENG can be flexibly constructed from a 1D structure into 2D or 3D forms, demonstrating good structural scalability [71, 72, 73, 74]. Stretchable FS‐TENGs are typically designed with single‐electrode or coaxial double‐electrode contact‐separation modes. The single‐electrode FS‐TENG generates electrical signals through contact with external objects, while the coaxial double‐electrode FS‐TENG makes contact when stretched to a certain length [75, 76, 77, 78, 79, 80]. Additionally, a helical structure can be built on a stretchable fiber substrate, allowing the fabrication of a helical fiber strain sensor (HFSS), as shown in Figure 4a [59]. PTFE/Ag and nylon/Ag woven fibers are alternately wound on the stretchable substrate to form the HFSS. During the stretching and releasing process, the helical structure extends or retracts longitudinally, driving periodic contact‐separation between the PTFE and nylon layers. Even with minimal stretching, the contact state changes, generating a stable electrical signal, with strain detection capabilities below 1%. The HFSS can be integrated into a chest strap and fixed below the chest, continuously monitoring respiratory movements caused by chest deformations through signal processing. As shown in Figure 4b, the HFSS can clearly distinguish between normal, rapid, and deep breathing patterns. Rapid breathing generates more voltage output peaks (*V*_oc_) per unit time, with a frequency of about 54 times/minute, significantly higher than the 20 times/minute of normal breathing [59]. In contrast, deep breathing has larger *V*_oc_ amplitudes and wider peaks, indicating higher intensity but lower frequency.

For FS‐TENG, pressure sensing is predominantly facilitated through the tensile mode, which is intrinsically linked to the linear macroscopic and microscopic structures of the fiber [81]. Therefore, FS‐TENG is mainly suitable for sensing tensile forces along the fiber's axis [78, 79, 80, 82, 83]. In contrast to the energy conversion efficiency issues caused by the small fiber contact area [84], textile‐based TENGs, due to the 2D flexible structure of the fabric, provide sufficient surface contact area and greater flexibility in terms of the direction of applied force [40, 85]. At the same time, due to the diversity of materials and structural flexibility, textile fabrics have become ideal wearable TENG carriers. Figure 4c demonstrates the interlock stitch knitted TENG (ISK‐TENG) based on textile fabric and its yarn structure [40]. The fabric is woven by alternating PTFE monofilament with cotton/silver‐plated PA6 coated yarn, where the latter is made by winding silver‐plated PA6 multi‐strand yarn around a cotton core yarn. The two yarns form a pipeline‐like structure arranged in parallel, providing ample space for triboelectric charge accumulation during the deformation process of the fabric. By securely integrating the ISK‐TENG into a tight garment and attaching it to the chest, efficient respiratory monitoring can be achieved, as shown in Figure 4d,e, which correspond to slow breathing at rest and rapid breathing after exercise [40]. In addition to applications in textile fabrics, TENG can also be combined with soft materials such as latex and silicone rubber to fabricate flexible TENGs. As shown in Figure 4f, a flexible waterproof fish lateral line system fiber‐based TENG (FLLF‐TENG) is demonstrated [38]. Its structure simulates the fish's lateral line canal using latex balloon‐wrapped airways, while helical silver‐plated nylon yarn mimics sensory nerves, and porous silicone rubber and nylon fluff simulate the hair cells at the top of the lateral line neuromast. Under external load, the nylon fluff and porous silicone rubber approach each other, and the FLLF‐TENG demonstrates good flexibility and output performance, with the ability to quickly recover its shape. The FLLF‐TENG can be elastically combined with a band and fixed to the subject's abdomen, sewn with yarn, and both ends secured with black tape. During inhalation, the chest and abdomen expand, compressing the FLLF‐TENG and generating electrical signals. During exhalation, the external pressure decreases, and the internal pressure of the balloon causes the friction layers to separate, generating opposite electrical signals. Through the inhalation and exhalation cycles, the FLLF‐TENG can accurately output voltage signals for three different breathing patterns (Figure 4g) [38]. Research on TENGs based on film and fiber structures provides a new framework for the development of biosensing technology and smart textiles, with its universality and scalability opening up broad application prospects in the field of respiratory health monitoring.

## 4 Respiration Monitoring via Piezoelectricity

Piezoelectric energy harvesting devices have attracted widespread attention due to their ability to convert environmental mechanical energy into electrical energy [86, 87, 88]. The PENG based on ZnO nanowires was reported in 2006 [89], marking a foundational breakthrough that initiated the development of PENG technologies. Following this pioneering work, the field experienced steady expansion, with advances in material synthesis, device miniaturization, and structural engineering gradually shaping PENGs into a versatile platform for energy harvesting and self‐powered active sensing. PENGs have been applied in various fields, especially in healthcare, for real‐time monitoring of human movement and physiological signals to assist in health assessment [90, 91, 92]. PENGs can generate electricity from small deformations and have advantages such as low cost, small size, and ease of integration [93]. Micro/nanofiber fabrics, owing to their excellent mechanical flexibility, are often used as supporting substrates for piezoelectric nanomaterials, enabling stable energy conversion under various mechanical stimuli. Embedding piezoelectric ceramics such as ZnO, BaTiO_3_, and SbSI into organic fibers like cellulose and nylon has been successfully applied in sensing, driving, and energy harvesting [89, 94, 95, 96, 97, 98].

**FIGURE 5:**
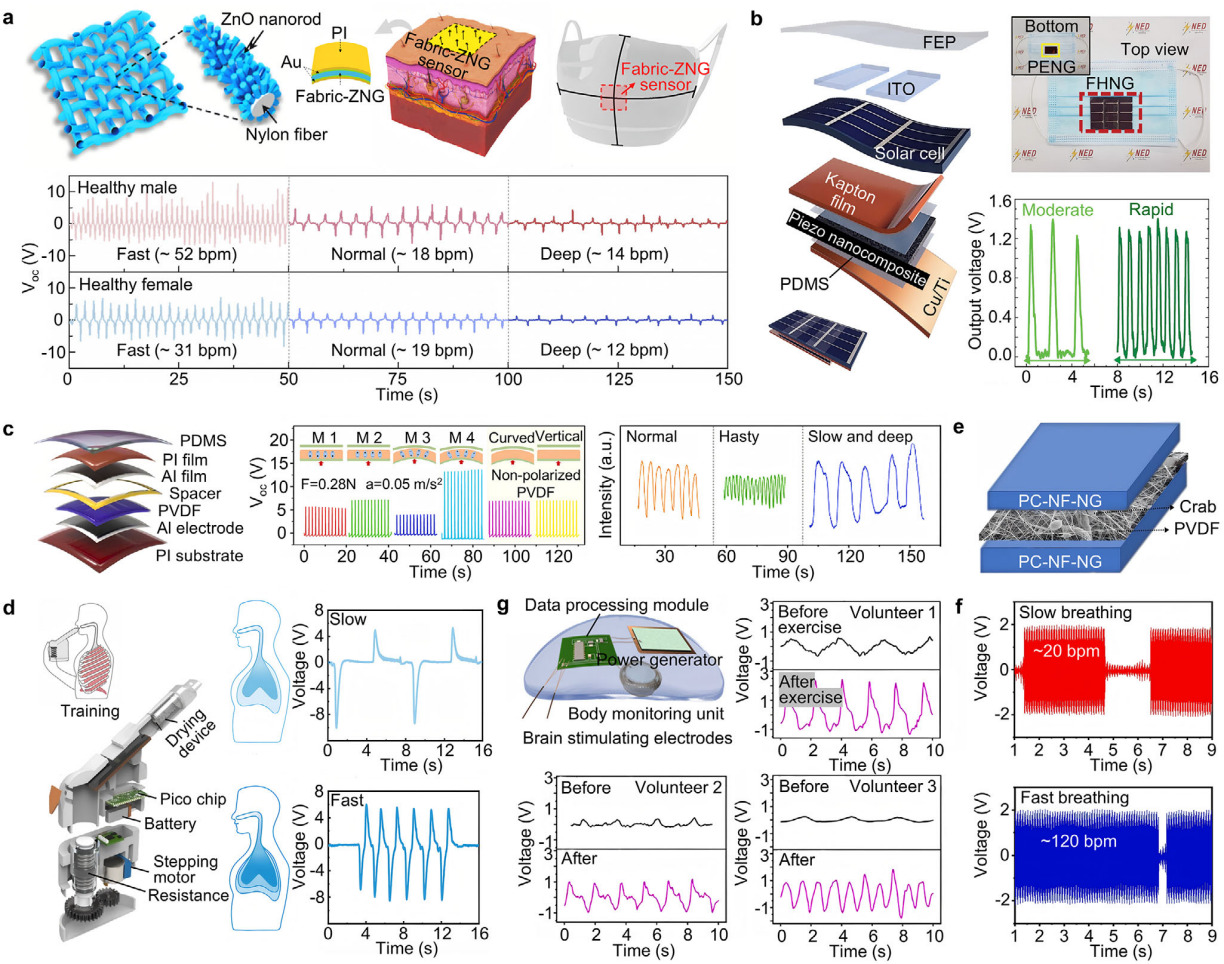
Applications of piezoelectric nanogenerators for respiratory rate monitoring. (a) Schematic diagram of fabric and piezoelectric ZnO nanogenerators (fabric‐ZNG); fabric‐ZNG‐based sensor implemented on a human skin interface; sensor attachment to a face mask for detecting breathing through nose and mouth and pyroelectric current‐sensing results for a healthy male and female under different breathing modes, including fast, normal, and deep breathing [108]. Adapted with permission from ref. [108]. Copyright 2023 ELSEVIER. (b) Schematic of the flexible hybrid nanogenerator (FHNG); photograph of the wearable FHNG for detecting human oral respiration and output voltages of the wearable FHNG for detecting the human oral respiration under the different respiration modes [109]. Adapted with permission from ref. [109]. Copyright 2021 Wiley‐VCH. (c) Schematic diagram of TENG based on Al and PVDF film; the output Voc of TENG driven by a linear motor in different modes and the respiration signals [62]. Adapted with permission from ref. [62]. Copyright 2022 ELSEVIER. (d) Schematic diagram of the intelligent respiratory trainer and voltage signal diagram of slow respiratory and fast respiratory [114]. Adapted with permission from ref. [114]. Copyright 2025 Wiley‐VCH. (e) Schematic representation of PVDF‐crab shell powder composite. (f) Application of the PENG as a respiratory sensor [115]. Adapted with permission from ref. [115]. Copyright 2024 ELSEVIER. (g) Four components of the system and the outputting piezoelectric voltage of respiration [122]. Adapted with permission from ref. [122]. Copyright 2021 ELSEVIER.

Currently, PENGs based on various piezoelectric materials have been developed to harvest energy from breathing and monitor respiratory rate [24, 99, 100, 101, 102, 103]. For example, a sensor combining polyvinylidene fluoride (PVDF) nanofibers and ZnO nanorods has been employed for harvesting respiratory energy [104]. In addition, PVDF nanofiber patches with piezoelectric fillers can monitor respiratory rate and patterns, distinguishing between normal and abnormal breathing [105]. Expanding on this capability, highly sensitive PVDF nanofiber sensors can simultaneously monitor both respiration and facial activities [106]. Furthermore, respiratory masks containing ZnO nanoparticles exhibit high sensitivity to breathing signals, making them well‐suited for wearable respiratory monitoring [107]. The upper part of Figure 5a shows a wearable sensor based on nylon fabric and piezoelectric ZnO nanogenerators (fabric‐ZNG), which features a hierarchical interlocking structure capable of withstanding various mechanical deformations such as twisting, wrinkling, and bending, ensuring reliable biological monitoring [108]. Due to its bending characteristics, this sensor can respond in real time to skin surface deformations caused by muscle contraction and relaxation. The lower part of Figure 5a displays the measurement results of fabric‐ZNG installed on a mask near the wearer's mouth and nose, showing good differentiation for different breathing patterns (such as rapid, normal, and deep breathing), demonstrating its excellent recognition capability [108].

PENGs can be integrated with TENGs, expanding their applications in portable devices and biosensors. Figure 5b shows a flexible hybrid nanogenerator (FHNG) consisting of TENG, solar cells, and PENG, featuring an ultra‐thin and flexible design that easily deforms under small impacts [109]. The TENG is based on a fluoroethylene propylene (FEP) film and indium tin oxide (ITO) electrodes, capable of harvesting electrostatic energy from raindrop motion. The structure of the PENG is top electrode/dielectric layer/p‐NC layer/dielectric layer/bottom electrode, and it can harvest energy from raindrop impact, wind energy, and human mechanical energy. When integrated into a commercial mask, the FHNG can detect oral breathing frequency, generating an output voltage of 1.4 V during exhalation and enabling the perception of different oral breathing frequencies by measuring the output performance during moderate and rapid exhalation [109]. In addition to being integrated with TENG, piezoelectric materials can significantly enhance the electrical output of TENGs. PVDF and its copolymers PVDF‐TrFE and PVDF‐HFP possess excellent piezoelectric properties, mechanical stability, processing flexibility, and cost‐effectiveness, making them ideal piezoelectric composite fiber substrates that combine strength, flexibility, and moldability [110, 111, 112]. Figure 5c shows a contact‐separation mode TENG based on a multilayer structure, consisting of two parts: a polarized PVDF film with an aluminum back electrode and an aluminum film deposited on a PI film substrate [62]. The surface of PVDF is more prone to capturing electrons than aluminum, and there is a hollow rubber spacer between the PVDF and aluminum to maintain separation. Experiments show that the polarization direction of PVDF and the working mode significantly affect the electrical output of TENG, and the piezoelectric electronic effect can improve the performance of TENG‐based pressure sensors by adjusting the interface energy barrier, achieving high sensitivity and linear response across a wider detection range. The PVDF‐based TENG exhibits significant signal differences during normal, rapid, and slow diaphragmatic breathing, proving that the sensor's voltage signal can accurately reflect the depth and rate of breathing [62]. In addition to multi‐scenario applications and performance enhancement, PENG can also combine well with TENG based on measurement characteristics. Compared to TENG, PENG is more sensitive to low‐frequency deformation, making it suitable for low‐frequency signal detection [113]. Figure 5d shows a TENG–PENG hybrid sensor that can simultaneously sense high and low‐frequency signals, identify various breathing patterns, and improve detection accuracy through dual channels. It effectively records breathing depth and speed and remains stable after 5000 airflow cycles, demonstrating excellent durability [114].

PENGs can also be integrated with natural biodegradable materials to achieve enhanced biocompatibility and improved output performance. Figure 5e shows a low‐cost, environmentally friendly PENG prepared by combining crab shell waste with PVDF [115]. Crab shell powder, rich in chitosan and calcium carbonate, is electrospun and compounded with PVDF to form nanocomposites with good mechanical and piezoelectric properties. The structural irregularities introduced by crab shell nanofibers enhance the deformation of the matrix under stress, promote polarization, and generate surface charges at the interface, while the natural piezoelectricity of chitosan further improves the response performance [116, 117]. The PENG fabricated from PC‐NFs exhibits good mechanical stability and sensitivity, accurately distinguishing slow and fast breathing (Figure 5f) [115]. In addition to the integration with new materials, PENG can further expand in application scenarios. In recent years, wearable brain–machine interfaces have shown great promise in assisting physical exercise, and self‐powered systems can continuously monitor vital signs, enabling the comprehensive collection and processing of training data [118, 119, 120, 121]. Figure 5g shows a self‐powered wearable brain–machine interface system for improving sports endurance performance. This system, based on a flexible substrate, includes a power generator, body monitoring unit, data processing module, and brain stimulating electrodes, while the detector consists of piezoelectric PVDF film, Ag electrodes, PET film, and a 3D‐printed flexible resin shell. After being attached near the chest, the system can monitor the respiration rate of subjects before and after exercise, clearly showing differences in breathing patterns, depth, and frequency among three subjects [122]. In summary, PENGs have exhibited strong potential in advancing technologies for respiratory health monitoring.

## 5 Respiration Monitoring via Magnetoelasticity in Soft Matter

**FIGURE 6:**
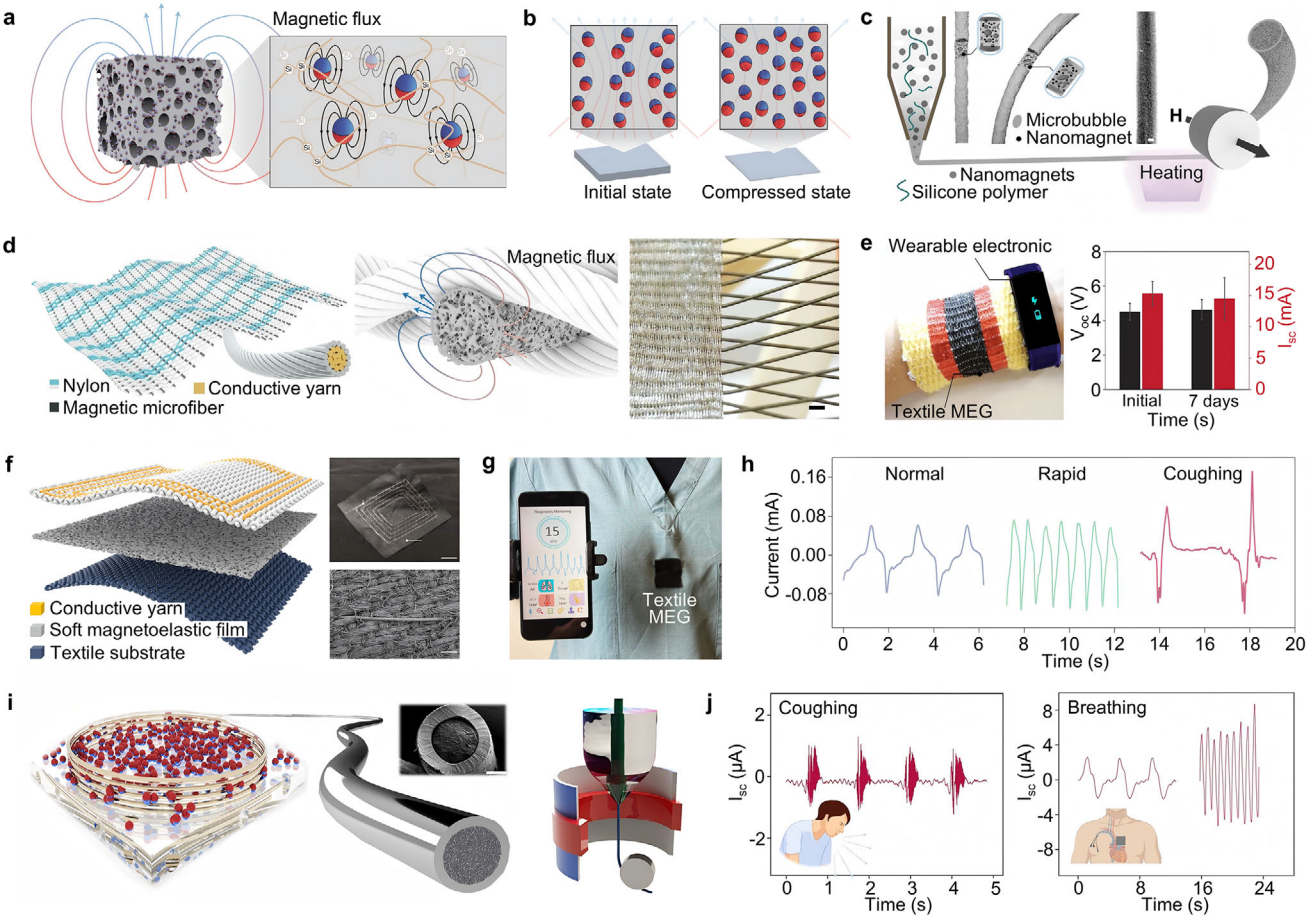
Applications of magnetoelastic generators in respiratory rate monitoring. (a) Sketch of the porous, soft system generating a magnetic flux. (b) Illustration of the magnetic dipole alignment changing the magnetic flux density of the soft system in the initial state and the compressed state based on a wavy chain model, red and blue spheres represent micromagnets [27]. Adapted with permission from ref. [27]. Copyright 2021 Springer Nature. (c) Schematic of the soft magnetic fiber fabrication process; Micro‐computed tomography (micro‐CT) image and schematics of the soft magnetic fiber. (d) Schematic design of the textile MEG; schematics of the working mechanism of the textile MEG, and a photograph showing the weaving processing with a loom. (e) Schematic of the textile MEG mixed with wool fibers driving a wearable biosensor system and electric outputs of the textile MEG before and after being submerged in water for 7 days [28]. Adapted with permission from ref. [28]. Copyright 2021 Springer Nature. (f) Textile MEG, photograph of the scalable textile coil, and SEM of the textile coil. (g) Textile MEG integrated on the nursing scrubs for respiratory monitoring. (h) Three different kinds of respiratory patterns: normal breathing, rapid breathing, and coughing were monitored by the textile MEG [29]. Adapted with permission from ref. [29]. Copyright 2021 ELSEVIER. (i) Diagram of the magnetoelastic sensor and SEM image of the cross‐section of the liquid metal microfibers and schematic setup of the thermal fiber drawing tower. (j) Current output associated with coughing, hyperpnea, and tachypnea [30]. Adapted with permission from ref. [30]. Copyright 2022 ACS.

Leveraging the giant magnetoelastic effect in soft matter that was discovered in 2021 [27], showing a magnetomechanical coupling factor up to four times higher than that of conventionally observed in rigid metal and metal alloys [27, 123], MEG devices transduce pressure‐induced magnetic‐field changes into robust signals, enabling water‐proof soft bioelectronics with multifaceted applications in biomedical monitoring [30, 124, 125, 126, 127, 128, 129, 130, 131, 132, 133], energy harvesting [134, 135, 136, 137, 138, 139, 140, 141], and therapeutic interventions [142, 143, 144] including respiration monitoring. As shown in Figure 6a, the soft magnetoelastic bioelectronics are constructed from a soft composite system consisting of micromagnets dispersed within a porous polymer matrix. Upon pulsed magnetization, the micromagnets form a wavy chain‐like alignment [27]. Unlike the conventional magnetoelastic effect, which conventionally relies on domain rearrangement and stress‐induced magnetic anisotropy under an external magnetic field [145], the giant magnetoelastic effect in MEGs originates from deformation‐induced changes in the micromagnet chain structure (Figure 6b). Mechanical compression alters the dipole–dipole interactions and demagnetizing fields within the chain; upon release of the applied stress, the chain structure recovers, and the magnetic flux density returns to its original state [27]. Figure 6c illustrates the fabrication process of a soft magnetoelastic fiber for wearable textile bioelectronics based on MEGs [28]. A ternary mixture is first prepared by dispersing magnetic nanoparticles into high‐viscosity liquid silicone and introducing air microbubbles, followed by extrusion and thermal crosslinking to form soft magnetic fibers with tunable diameters. Micro‐computed tomography (Micro‐CT) imaging reveals uniformly distributed nano‐ to microscale internal cavities, contributing to excellent flexibility and deformability. Once formed, the soft magnetic fibers are woven with silver‐coated nylon and conventional nylon‐conductive yarns to construct the textile MEG, which is compatible with standard loom‐based mass production (Figure 6d). In this system, mechanical deformation induces variations in the magnetic field, which are converted into electrical energy via induction in the conductive yarns. The textile MEG can be worn on a perspiring human arm (Figure 6e) to power integrated wearable biosensing systems for real‐time physiological monitoring. Notably, the unencapsulated textile MEG remains fully functional even after one week of immersion in water or operation in high‐humidity environments [28].

The textile‐based MEG demonstrates intrinsic waterproof capability even without encapsulation, as magnetic fields can propagate through water molecules with minimal attenuation of intensity. This property renders it particularly suitable for self‐powered respiratory monitoring in high‐sweat environments (Figure 6f) [29]. As shown in Figure 6g, the textile MEG can be seamlessly integrated into garments, offering high breathability and wearing comfort. It enables continuous respiratory monitoring even in sweat‐intensive regions such as the chest. When worn by a subject, the device successfully captured distinct waveforms corresponding to normal breathing, rapid breathing, and coughing (Figure 6h), highlighting its high sensitivity and stability in detecting variations in frequency, amplitude, and duration across different respiratory patterns. Beyond textile architectures, MEGs can also be used to develop stretchable biomedical sensors by incorporating liquid metal fibers for respiratory rate monitoring. Figure 6i presents an ultrastretchable pressure sensor based on MEG technology [30], fabricated by thermally stretching styrene‐ethylene‐butylene‐styrene (SEBS)‐covered poly(vinyl alcohol) (PVA) rods to form elastic microtubes. Liquid metal alloy is then injected into the hollow channels to create liquid metal microfibers, which are coupled with soft polymer composites to construct a highly stretchable film. This magnetoelastic sensor, when applied to the skin surface, can accurately monitor respiratory behaviors such as coughing, deep breathing, and rapid respiration—even under heavy perspiration conditions (Figure 6j). In summary, owing to its exceptional moisture resistance, MEG represents a milestone in biomechanical energy harvesting and holds promise for broad applications in healthcare monitoring and soft robotics.

## 6 Comparison and Analysis

Advances in the three classes of self‐powered respiration monitoring technologies have created a growing need for a systematic comparison of representative devices based on key performance metrics, which enables a clearer understanding of the characteristics of different technological pathways. **Table** **1** summarizes the performance metrics of representative TENG, PENG, and MEG enabled devices for respiration monitoring, including sensitivity, detection range, stability, response time, and output power. Owing to the substantial differences in device structures and input forms, these parameters are not directly comparable on a quantitative basis, but they provide meaningful trends for qualitative assessment. Collectively, TENGs offer high structural designability and exhibit a broad and adjustable detection range that can span from 0%–80% strain to air‐flow rates of several hundred L/min, and generally maintain good stability [38, 40, 59, 64]. PENGs possess intrinsic advantages in capturing small pressures or subtle deformations, making them suitable for pressure‐oriented respiratory pattern recognition. They also demonstrate good stability and repeatability, and exhibit a response‐time range comparable to TENGs [62, 108, 109, 114, 122]. Benefiting from the giant magnetoelastic effect, MEGs achieve relatively balanced overall performance, with response times lying within the few‐millisecond range, detection ranges extending from the pascal to the megapascal regime, and consistently robust stability [29, 30]. With respect to the wearing comfort, fiber‐based, textile‐integrated, and thin‐film TENG and PENG structures offer favorable conformability, whereas MEG devices incorporating soft elastomers with embedded magnetic particles enable long‐term skin attachment and comfortable wear. Consequently, considering the combined factors of sensitivity, durability, and wearing comfort, MEGs offer the most favorable combination of functional characteristics among the three technologies. In terms of output power, the three types of devices span from the nanowatt level to the milliwatt level, as shown in Table 1. These values are jointly influenced by material systems, structural designs, load conditions, and transduction mechanisms, providing a general reference for the energy‐output characteristics of different sensing technologies.

**TABLE 1:** Comparison of key performance metrics of representative devices across the three technologies.

Technology type	Sensitivity	Detection range	Stability	Response time	Output power	Refs.
TENG	2.26 V/kPa	0–80 kPa	30 000 cycles	—	10.01 µW (160 MΩ)	[38]
TENG	—	0%–50% strain	10 000 cycles	120 ms	—	[40]
TENG	—	—	—	—	5.61 µW (35 MΩ)	[57]
TENG	—	—	500 cycles	—	1.2 µW (5 MΩ)	[58]
TENG	—	0%–80% strain	20 000 cycles	70 ms	4.3 µW/m (250 MΩ)	[59]
TENG	—	85–216 L/min	—	—	1.3 mW (15.1 MΩ)	[64]
TENG	—	—	—	—	192 nW (300 MΩ)	[66]
TENG	—	2–8 L/min	—	—	—	[67]
PENG	18.8 V/kPa	0.011–0.48 N	5000 cycles	72 ms	—	[62]
PENG	0.15 nA/kPa	3.07–827.6 kPa	500 cycles	69 ms	0.32 µW (20 MΩ)	[108]
PENG	—	—	160 000 cycles	—	88 mW/m^2^ (20 MΩ)	[109]
PENG	1.87 V/(m/s)	0−15 m/s	5000 cycles	160 ms	—	[114]
PENG	—	19.6–63 breaths/min	2000 cycles	—	82.95 µW (216 kΩ)	[122]
MEG	0.27 mA/kPa	0–6.5 kPa	5000 cycles	15 ms	0.405 mW (20 Ω)	[29]
MEG	—	3.5 Pa–2000 kPa	12 000 cycles	3 ms	—	[30]

In self‐powered respiration monitoring systems, the mechanical stability of devices under repeated deformation, humid environments, and long‐term skin attachment is essential for ensuring reliable signal acquisition. TENG, PENG, and MEG technologies all exhibit certain degrees of fatigue resistance and drift suppression through material and structural designs. For TENGs, fiber‐based and textile‐based structures exhibit robust durability over tens of thousands of cycles. Specifically, fiber‐shaped TENGs maintain stable performance after 20000 cycles [59], whereas yarn‐based, textile, and fabric‐integrated designs demonstrate consistent signal repeatability over approximately 10 000–30 000 mechanical cycles [38, 40]. In addition, in some TENG structures, flexible thin films undergo light‐contact, non‐adhesive oscillations under airflow, which reduces interfacial adhesion and mechanical abrasion, thereby improving long‐term output stability [64]. PENG devices benefit from the inherent electromechanical stability of mature piezoelectric materials such as PVDF and PZT, together with optimized configurations involving flexible electrodes and elastomeric substrates, thus exhibiting strong durability under repeated loading. Under various mechanical loading conditions, including 2000 bending cycles, 5000 airflow cycles, or 160 000 water‐droplet impacts, PENG devices continue to deliver stable outputs [109, 114, 122]. In comparison, MEGs show comparatively stronger stability. MEGs produce magnetic‐field variations through the magnetoelastic effect and transduce them via electromagnetic induction, without relying on interfacial charge accumulation or triboelectrification. As a result, their outputs remain essentially insensitive to sweat and humidity [30].

With respect to signal drift and calibration, multiple strategies that help suppress drift and reduce calibration burden have already emerged across device designs and signal‐processing methodologies. For TENGs, these strategies span materials, structures, and algorithms. Typically, in airflow‐driven tubular configurations, non‐adhesive oscillations generate stable periodic signals, thereby partially mitigating potential drift arising from fluctuations in interfacial contact states at the structural level [64]. In addition, in mask‐type configurations, the continuous accumulation of exhaled moisture leads to a gradually stabilized local humidity environment. Under this high‐humidity equilibrium, TENGs exhibit relatively consistent output amplitudes over time, which helps alleviate humidity‐induced drift and the associated calibration pressure [146]. On the signal‐processing side, digital filtering, baseline correction, and necessary normalization can be applied to extract respiratory features such as breathing frequency and amplitude from raw voltage waveforms. These features are then used for respiratory‐state classification via threshold‐based or pattern‐recognition strategies, thereby reducing the influence of noise and slow drift at the software level [147, 148]. For PENGs, structural strategies improve compliance to deformation, such as growing ZnO nanorod arrays on textile substrates to accommodate multidirectional bending and local respiratory perturbations at mask interfaces [108]. Electrospun PVDF can form conformal contact on the face or attachment sites, reducing waveform distortion due to loosening or local displacement, thereby improving long‐term signal consistency [115]. For MEGs, the adoption of well‐encapsulated magnetoelastic units and coil structures ensures that their outputs are minimally affected by environmental variations [29, 30]. Such environmental robustness reduces sensitivity to external humidity changes or fluctuations in attachment pressure, thereby decreasing drift‐inducing factors and alleviating repeated calibration demands during long‐term monitoring. Collectively, these strategies, spanning materials, structures, and signal‐processing approaches, form a diverse set of strategies for mitigating drift and calibration challenges, establishing an essential foundation for the long‐term operational stability of self‐powered respiration‐monitoring systems.

From material, structural, and mechanistic perspectives, TENGs, PENGs, and MEGs share different design principles across material combinations, interfacial configurations, and signal‐coupling modes, although all three technologies employ combinations of flexible substrates and functional units to enhance material compliance, interfacial conformity, and local microstructural modulation, enabling attachment to various body locations and accommodating diverse respiration‐related signals, such as thoracic deformation, local pressure variation, and airflow disturbances. For instance, TENGs often employ fiber‐based or textile‐integrated structures to enhance wearability and flexibility [38, 40, 59]. In PENGs, PVDF and its composite systems are widely used as piezoelectric materials, typically fabricated into flexible films and integrated with shells or attachment layers to achieve self‐powered respiratory‐state monitoring [114, 115, 122]. MEGs similarly rely on soft elastomers as substrates, within which magnetic particles form chain‐like microstructures, and external forces modulate the magnetic flux density through magneto‐mechanical coupling, enabling stable respiration‐related electrical outputs [29, 30]. Regarding innovation, each technology pathway continues to evolve through geometric optimization, material engineering, and cross‐mechanism integration to obtain more stable and wearable responses under low‐frequency deformation, large strains, or high‐humidity respiratory environments. However, evaluations of output reliability and long‐term stability under complex loading conditions and multi‐source interference remain limited. These challenges continue to drive the exploration of new structures and coupling mechanisms. In particular, high‐humidity environments represent a critical factor that compromises the output stability of TENGs and PENGs and are widely regarded as a potential barrier to commercialization [149, 150, 151]. A variety of humidity‐resistance strategies have been reported. For TENGs, approaches include employing encapsulation layers to reduce moisture ingress and leveraging surface engineering to construct hydrophobic, superhydrophobic, or moisture‐passivating interface [151, 152, 153, 154]. For PENGs, conventional PVDF‐based devices are prone to output degradation under sweat and moisture exposure due to water retention, whereas dense encapsulation significantly reduces breathability and comfort. Sweat‐permeable PVDF piezoelectric textiles alleviate this trade‐off to some extent [155]. Nevertheless, achieving an optimal balance between mechanical reliability and long‐term wearing comfort remains an open challenge in the broader design of wearable piezoelectric textiles. Among these challenges, humidity sensitivity remains a key engineering issue requiring further optimization, and its limitations continue to motivate ongoing research. From an evolutionary perspective, current technology development is advancing along several directions. First, multimodal fusion is emerging as an important design paradigm, enabling devices to capture multiple respiration‐related features simultaneously and thereby enhance signal‐interpretation capability [114]. Second, device structures are transitioning from traditional thin‐film formats to fiber‐based and textile systems, yielding significant improvements in stretchability, breathability, and integrability [29, 38, 64]. Third, the introduction of new self‐powered mechanisms, particularly those based on magnetic‐field coupling, provides alternative sensing pathways for respiration monitoring and ensures stable magnetic‐flux modulation under high humidity, sweat exposure, or interfacial disturbances [29, 30]. These performance characteristics shape device stability in complex environments while also determining their suitability across diverse application scenarios. Clinical respiratory assessment, continuous home monitoring, and motion‐state tracking typically require lightweight wearable sensors capable of stable long‐term operation without external power sources. Self‐powered respiration sensors thus exhibit clear advantages in these contexts.

## 7 Conclusion and Future Perspectives

This work reports the research progress in self‐powered sensors for respiratory rate monitoring, focusing on the working mechanisms of using TENGs, PENGs, and MEGs. It further synthesizes the comparative performance characteristics, stability differences, and application suitability of these three technologies, outlining their shared design principles and emerging trends. TENGs generate static charges through the triboelectric effect and electrostatic induction [43, 64]. Due to their high sensitivity, low cost, self‐powering capability, small size, and excellent stretchability, TENGs have been widely used in the development of respiration monitoring sensors [58, 156, 157, 158, 159, 160]. PENGs, on the other hand, generate electrical energy under small deformations [48, 49] and also have advantages such as low cost, compact size, and ease of integration [93], making them widely applied in respiratory rate monitoring [25, 108, 161]. TENGs and PENGs generally achieve multi‐scenario applications through different structural designs, and their performance can be enhanced by combining both [62, 114]. When applied to the skin surface or in high‐humidity environments, the sweat on the skin surface affects their output performance and significantly impacts their stability [51, 52]. Encapsulation can reduce the impact of moisture to some extent, but it can also affect the comfort of wearing the sensor and its performance. MEGs, however, address the waterproof issue by principle. Even without encapsulation, MEGs are completely waterproof due to their reliance on the magnetoelastic effect, where the magnetic field can pass through water molecules with almost no loss of strength. This makes MEGs capable of wireless self‐powered respiratory monitoring in humid environments while maintaining flexibility and stretchability, and they can accurately monitor and distinguish multiple breathing patterns even during heavy sweating [29, 30], greatly compensating for the lack of waterproofing in other generators.

**FIGURE 7:**
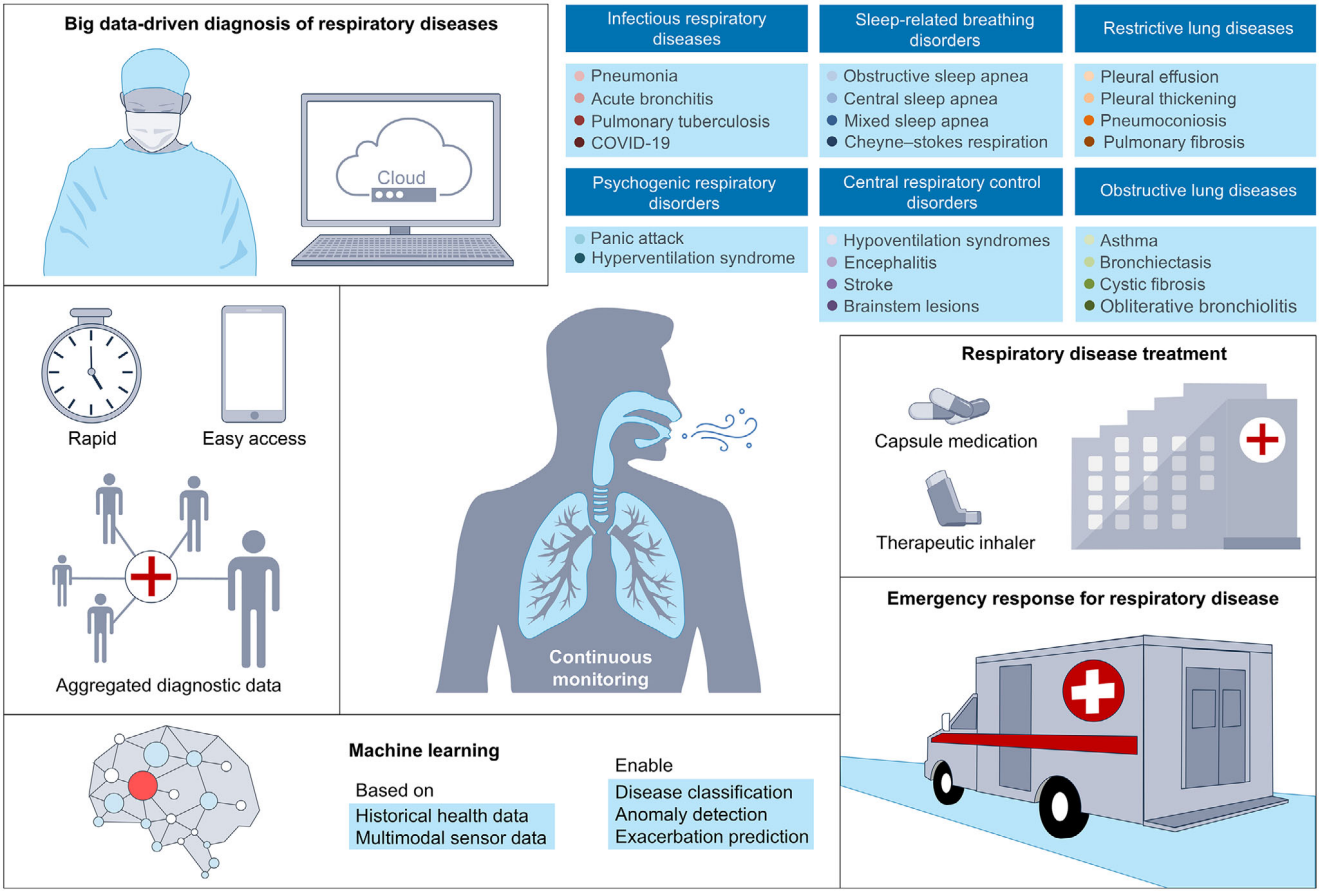
Perspectives regarding self‐powered respiratory rate monitoring. Respiration monitoring bioelectronics based on TENGs, PENGs, and MEGs offer continuous monitoring capabilities, enabling real‐time transmission of collected respiratory data to cloud platforms for centralized aggregation and storage. This supports the development of big data‐driven diagnostic paradigms, facilitating remote diagnosis, the construction of electronic health records, and auxiliary diagnosis based on historical health data. Patients can thereby gain faster and more convenient insights into their health status. In addition, machine learning can effectively identify respiratory features associated with various diseases. Respiratory‐related conditions such as obstructive sleep apnea, pneumonia, asthma, acute bronchitis, and COVID‐19 can be assessed and predicted through big data analysis. Furthermore, by integrating the collected and diagnosed data with medical treatment protocols, patients can be provided with precise medications and therapeutic devices. In cases of severe respiratory emergencies, emergency services can be promptly dispatched to ensure rapid medical intervention.

The above‐mentioned self‐powered sensors possess continuous monitoring capabilities. By integrating big data‐driven diagnosis, especially assisted diagnosis based on historical health data, it is possible not only to achieve precise disease diagnosis for individuals but also to facilitate early detection and timely intervention of various respiratory diseases (Figure 7). Looking ahead, continued advances in nanomaterials, biomedical engineering, the Internet of Things, and machine learning are expected to further propel the development of self‐powered respiration monitoring in several key directions.

### 7.1 Long‐Term and Multi‐Scenario Respiratory Data Collection

The practicality of the sensors must meet the requirement for stable operation in different environments (such as nighttime sleep, outdoor exercise, high humidity, high temperature, etc.). Therefore, future research should focus on improving the energy output durability, anti‐interference, and stability of the generators and sensors, as well as developing sensing systems with dynamic adaptive capabilities. For example, designing respiratory monitoring sensors with temperature compensation and motion artifact elimination systems [162, 163] will ensure continuous, high‐quality respiratory data collection during the user's daily life.

### 7.2 Big Data‐Driven Diagnosis

With the support of IoT and 5G communication technologies, respiratory data collected from wearable devices can be transmitted in real‐time to cloud platforms, enabling seamless data aggregation and storage [164, 165, 166]. This big data‐driven diagnostic paradigm enables remote diagnosis, electronic health record construction, assisted diagnosis based on historical health data, and cross‐regional sharing of medical resources, as illustrated in the upper left and middle images of Figure 7. Based on the respiratory data collected online, doctors can provide personalized medical advice to patients [167], while patients can easily and quickly understand their health status. As the amount of data increases, relevant data can be aggregated to build electronic health records and clinical databases, providing solid data support for the subsequent development of personalized respiratory disease screening and evaluation models. This process advances respiratory monitoring and treatment from intermittent recording and diagnosis toward continuous tracking, high‐precision diagnosis, and data‐driven personalized therapy.

### 7.3 Machine Learning‐Based Respiratory Disease Identification

By incorporating deep learning, pattern recognition, and feature extraction algorithms into the analysis of respiratory data, it is possible to effectively identify changes in respiratory characteristics under different disease conditions, as shown in the lower left image of Figure 7. Various respiratory‐related diseases, including obstructive sleep apnea [168], pneumonia [169], asthma [170], acute bronchitis [171], and COVID‐19 [19], can be assessed and predicted after big data analysis. Future research on big data‐driven diagnosis could further explore multimodal sensor data fusion (such as electrocardiogram, blood oxygen, skin temperature, etc.) to enhance the accuracy and robustness of respiratory disease identification.

### 7.4 Responsive Therapeutic Systems

While continuously monitoring, self‐powered respiratory rate sensors also provide the potential for timely responses in disease treatment. By combining the collected and diagnosed data with hospital treatments, it is possible to accurately provide patients with the necessary medication and therapeutic equipment. In the case of severe respiratory emergencies (such as acute respiratory distress syndrome, asthma attacks, etc.) [170, 172, 173, 174], the continuously monitoring respiratory sensors can transmit data in real‐time to hospitals. Hospitals can provide timely temporary rescue guidance based on the changes in data, and if necessary, arrange for an ambulance to immediately provide emergency treatment (Figure 7, right image), ensuring that patients receive the fastest medical response.

Self‐powered biosensors are rapidly advancing in the field of respiratory monitoring, holding the potential to transform traditional respiratory disease management based on typical intervention through big data‐driven diagnosis. In the future, through deep interdisciplinary integration, especially with continuous breakthroughs in material optimization, integrated manufacturing, machine learning algorithms, and system platform integration, their practical application in clinical diagnosis and home healthcare management will be accelerated, paving the way for precision medicine.

## Author Contributions

J.C. supervised the project. X.Xu., J.C., X.Xiao., and R.G. prepared the manuscript.

## Conflicts of Interest

The authors declare no conflicts of interest.
